# Aberrant Lipid Metabolism: An Emerging Diagnostic and Therapeutic Target in Ovarian Cancer

**DOI:** 10.3390/ijms14047742

**Published:** 2013-04-10

**Authors:** Carmen E. Pyragius, Maria Fuller, Carmela Ricciardelli, Martin K. Oehler

**Affiliations:** 1 Discipline of Obstetrics and Gynaecology, School of Paediatrics and Reproductive Health, Research Centre for Reproductive Health, Robinson Institute, University of Adelaide, Adelaide 5005, Australia; E-Mails: carmen.macsai@adelaide.edu.au (C.E.P.); carmela.ricciardelli@adelaide.edu.au (C.R.); 2 Genetics and Molecular Pathology, SA Pathology, Women’s and Children’s Hospital, Adelaide 5006, Australia; E-Mail: maria.fuller@adelaide.edu.au; 3 Department of Gynaecological Oncology, Royal Adelaide Hospital, Adelaide 5000, Australia

**Keywords:** ovarian cancer, lipid synthesis, signalling pathways, biomarkers, therapeutic targets

## Abstract

Ovarian cancer remains the most lethal gynaecological cancer. A better understanding of the molecular pathogenesis of ovarian cancer is of critical importance to develop early detection tests and identify new therapeutic targets that would increase survival. Cancer cells depend on *de novo* lipid synthesis for the generation of fatty acids to meet the energy requirements for increased tumour growth. There is increasing evidence that lipid metabolism is deregulated in cancers, including ovarian cancer. The increased expression and activity of lipogenic enzymes is largely responsible for increased lipid synthesis, which is regulated by metabolic and oncogenic signalling pathways. This article reviews the latest knowledge on lipid metabolism and the alterations in the expression of lipogenic enzymes and downstream signalling pathways in ovarian cancer. Current developments for exploiting lipids as biomarkers for the detection of early stage ovarian cancer and therapeutic targets are discussed. Current research targeting lipogenic enzymes and lipids to increase the cytotoxicity of chemotherapy drugs is also highlighted.

## 1. Introduction

Ovarian cancer is the most lethal gynaecological cancer and ranks as the fourth most common cause of cancer-related death in women in the Western world [1]. The poor prognosis is largely attributed to a lack of early detection and, consequently, approximately 70% of patients present with advanced-stage metastatic disease. The treatment for advanced ovarian cancer is debulking surgery followed by platinum- and taxane-based chemotherapy. This standard treatment results in a complete response rate of 40%–60%; however, more than 90% of patients relapse after 18 months and, with the emergence of chemoresistance, ultimately die from the disease [2]. Significant improvements in ovarian cancer survival will require the development of more effective molecularly targeted diagnostics and/or therapeutics.

In recent years, the field of cancer cell metabolism has been progressing rapidly. Cancer cells rely on *de novo* lipid synthesis for the generation of fatty acids to meet the needs of tumour growth, resulting in specific alterations in different aspects of lipid metabolism. These alterations can influence the availability of structural lipids for the synthesis of membranes, the production and degradation of lipids for energy supply and the abundance of lipids with signalling functions. Altered lipid metabolism is detected in ovarian cancer patients during early and late stages of disease, including patients with recurrent disease, when compared to healthy controls [3,4]. Consequently, there is convincing evidence to support a pivotal role of increased lipid synthesis in the development of ovarian cancer. The main classes of lipids implicated in the pathogenesis of ovarian cancer include the phospholipids and sphingolipids. Multiple pathways are involved in the synthesis and degradation of these lipid groups, involving changes in the expression and activity of enzymes regulating their metabolism.

This article reviews the latest knowledge on lipid metabolism in ovarian cancer cells and the alterations in the expression of lipogenic enzymes and signalling pathways. Current developments in the application of lipids as biomarkers in the detection of early stage ovarian cancer are discussed. Furthermore, research employed to overcome chemotherapy resistance by targeting lipogenic enzymes and lipids to increase the cytotoxicity of chemotherapy drugs is also highlighted.

## 2. Lipid Metabolism in Ovarian Cancer

Abnormal lipid metabolism, leading to increased lipid synthesis, is found to play an important role in the pathogenesis of malignancies, including ovarian cancer [5]. Cancer cells depend on *de novo* lipid synthesis for the generation of fatty acids to meet the needs of tumour growth [6]. The increased expression and activity of lipogenic enzymes is largely responsible for increased synthesis of long-chain fatty acids in cancer cells [7,8]. Changes in the expression and activity of enzymes involved in lipid metabolism are regulated by metabolic and oncogenic signalling pathways [9,10]. Common enzymes identified in a variety of human malignancies include ATP citrate lyase (ACL), acetyl-CoA carboxylase (ACC) and fatty acid synthase (FAS) [7,11,12]. These enzymes are essential in the biosynthesis of fatty acids, converting cytosolic citrate to acetyl-CoA (the precursor for fatty acids and cholesterol), followed by the production of malonyl-CoA, which is further converted to produce fatty acids, respectively. The expression and activity of ACL, ACC and FAS are regulated by complex interactions between numerous signalling pathways. For example, the phosphatidylinositol 3-kinase-Akt (PI3K-Akt) and LKB1/AMPK signalling pathways can increase enzyme activity via direct phosphorylation or by sterol regulatory element-binding protein (SERBP)-mediated transcription of lipogenic genes [9,10,13]. Other signalling components, including oncoproteins and tumour suppressors, regulate fatty acid synthesis, leading to alterations in the production of fatty acid precursors generated from both glucose and glutamine metabolism. In particular, tumour suppressor p53 regulates fatty acid synthesis via the pentose phosphate pathway (PPP) and suppresses glucose consumption, NADPH production and biosynthesis [14]. The PPP is also regulated by a target protein of p53, TIGAR, which decreases levels of fructose-6-phosphate, resulting in an inhibition of glycolysis and fatty acid synthesis [15]. p53 can also increase fatty acid synthesis via the enhanced expression of glutaminase 2 [16]. The increased synthesis of long-chain fatty acids provides cancer cells with the structural building blocks, signalling molecules, post-translational modification of proteins and energy required for membrane synthesis, cell survival, proliferation, migration and invasion, which are important for the initiation and progression of tumours [17].

Normal cells acquire fatty acids to meet their metabolic demands from exogenous sources, including dietary lipids. A high intake of dietary lipids, including food with a high level of saturated fat or cholesterol, such as red meat, eggs and dairy products, is a well a known risk factor for ovarian cancer [18–20]. A higher consumption of fats and cholesterol can increase the risk of ovarian cancer through increased levels of circulating estrogen and/or progesterone [21]. The exposure of the ovarian epithelium to these hormones during ovulation may contribute to malignant transformation and tumour development [21,22]. Nonetheless, the increased rate of *de novo* lipid synthesis that occurs during oncogenesis to meet the needs of tumour growth is shown to be irrespective of the nutritional load [23]. Interestingly, adipocyte derived lipids were shown to act as an energy source for ovarian cancer cells, and co-culture with adipocytes resulted in the transfer of lipids to ovarian cancer cells, promoting tumour growth *in vitro* and *in vivo*[24].

### Fatty Acid Synthase (FAS)

The increased expression of fatty acid synthase (FAS) is associated with a poor prognosis in a variety of human malignancies, including ovarian cancer [7,25]. The correlation between elevated FAS and enhanced tumour growth is attributed to the role of FAS activity in phospholipid synthesis. FAS activity is shown to drive phospholipid synthesis in the endoplasmic reticulum (ER), promoting ER homeostasis and, consequently, cell survival [26]. Treatment of a variety of cell lines with FAS inhibitors induces ER stress in tumour cells, inducing cell death [27] and inhibiting fatty acid synthesis [28]. Treatment of SKOV3 human ovarian cancer cells with a synthetic FAS inhibitor (C93) led to the activation of AMP-activated protein kinase (AMPK) and cell death [29]. Treatment of SKOV3 xenograft bearing mice with C93 also had a significant anti-tumour effect, causing a reduction in both tumour growth and volume [29]. The FAS inhibitor C75 has recently been shown to significantly reduce cell proliferation and induce apoptosis in ovarian clear cell carcinoma cell lines [30] and was attributed to the downregulation of the oncogenic phosphoinositide-3-kinase (PI3K) signalling pathway [31].

## 3. Lipids

### 3.1. Phospholipids

Phospholipids are commonly associated with cancer and have been identified in almost every type of malignancy [32]. Phospholipids are a major component of all cell membranes, spontaneously forming lipid bilayers. Lysophosphatidic acid (LPA) is a lysophospholipid that functions as an extracellular signalling molecule and mediates a variety of biological processes by binding to a family of cell surface G protein-coupled receptors [33]. These include LPA receptors belonging to the endothelial differentiation gene (Edg) family (LPA_1_/Edg2, LPA_2_/Edg4, LPA_3_/Edg7) [33], the purinergic family (GPR23/P2Y9/LPA_4_) [34] and the related receptors, GPR92/LPA_5_[35] and P2Y5/LPA_6_[36]. Receptors from the purinergic family and related receptors are structurally different to those from the well-characterised LPA_1–3_ receptors [34,37]. LPA receptors, in particular LPA_2,_ are shown to play a significant role in the pathogenesis of a variety of human malignancies, including epithelial ovarian cancer (EOC) [38,39].

LPA is secreted by several cell types, detected in many biological fluids, and stimulates cancer cell proliferation, migration and survival [40]. High levels of LPA are produced by ovarian cancer cells [40–42]. LPA is also elevated in the ascites of ovarian cancer patients [42,43]. It was recently demonstrated that LPA in EOC ascites is an important mediator of tumour-promoting effects in conjunction with phospholipase A2 (PLA_2_) activity (discussed below) [44].

Several studies have demonstrated that LPA activates proteolytic enzymes, including urokinase plasminogen activator (uPA) [45–47], matrix metalloproteinases (MMPs) MMP-2 [48] and MMP-9 [49] and VEGF [50–52], to augment ovarian cancer invasion and metastasis. The Ras/Rho/ROCK pathways are shown to contribute to LPA-induced proteolytic enzyme production [49,53,54]. LPA is shown to activate the Ras/Rho/ROCK pathways, to stimulate NF-kB, inducing the expression of MMP-9 and uPA, leading to the degradation of the basement membrane and various components of the extracellular matrix, respectively [49,55]. Furthermore, the LPA_2_ receptor was shown to be important in LPA-induction of uPA in ovarian cancer cells [46]. Inhibition of LPA_2_ receptor decreased LPA-induced uPA by over 50% and led to a reduction in invasion and migration of ovarian cancer cells [46]. Proteolytic enzyme production was also shown to be cell line-specific, where LPA induced the expression of uPA in SKOV-3 cells, but not MMP-9, whilst LPA induced the expression of MMP-9 in CAOV-3 cells [45,49]. Increased expression of VEGF results in increased angiogenesis and, therefore, increased tumour growth [50]. LPA stimulated the transcriptional activity of VEGF through c-Myc and Sp-1 independent of HIF-1α [52]. The Rho-ROCK pathway, which lies upstream of c-Myc, was also shown to contribute to LPA-induced VEGF expression [52].

LPA is also a major regulator of the chemokine growth-regulated oncogene α (GROα). GROα expression is identified in a number of human cancers and is associated with tumorigenesis, angiogenesis and metastasis [56–58]. More recently, elevated levels of GROα were found in plasma and ascites in a high percentage of ovarian cancer patients [59]. In ovarian cancer cell lines, GROα expression was not constitutive; however, strongly stimulated by LPA and mediated primarily by LPA_2_ receptors though the transcriptional activation of the GROα promoter [59]. A recent study demonstrated that LPA regulates the sterol regulatory element binding protein-FAS (SREBP-FAS) and AMP-activated protein kinase-ACC pathways (AMPK-ACC) resulting in increased *de novo* lipid synthesis in ovarian cancer cells [38]. In addition, LPA-induced lipid synthesis was mediated via LPA_2_ receptors [38], further supporting the importance of LPA_2_ signalling in the pathogenesis of ovarian cancer.

#### 3.1.1. Autotaxin (ATX)

Circulating LPA is primarily produced by autotoxin (ATX), a secreted enzyme, which has lysophospholipase D activity [60,61]. ATX is a member of the ectonucleotide pyrophosphatase and phosphodiesterase family of enzymes. It is a type II membrane protein synthesized as a secreted protein. ATX has lysophospholipase D activity, which enables it to convert lysophosphatidylcholine (LPC) into LPA [61]. Through its lysophospholipase D activity, ATX has been shown to act as a chemotactic factor, to stimulate angiogenesis [62] and to increase invasiveness and metastatic potential in transformed cells [63]. ATX was shown to be involved in ascites-induced migration of EOC [44]. Interestingly, although increased expression of ATX has been associated with increased cancer progression, ATX antigen levels in serum were not increased in ovarian cancer patients when compared to healthy controls [64]. Furthermore, ATX may not be useful as a diagnostic marker for ovarian cancer, as ATX is also elevated in other conditions, including liver disease, acute coronary syndrome and pregnancy [65–67].

#### 3.1.2. Phospholipase A

LPA is also produced by the actions of phospholipase A, PLA_1_ and PLA_2_[68]. High levels of cytosolic and calcium-independent PLA_2_ activity were found in human EOC ascites, concurrent with elevated levels of LPC, arachidonic acid, LPA and lipid products of PLA_2_[44]. Furthermore, a high level of PLA_2_ activity was observed in EOC tissues when compared to either benign or normal tissues [44]. PLA_2_ in ascites was also shown to induce the proliferation, invasion and migration of human EOC [44].

#### 3.1.3. Phospholipase D (PLD)

Phospholipase D (PLD) is an enzyme that converts membrane phospholipids to phosphatidic acid (PA), a precursor of LPA. PLD activity is increased in ovarian cancer cells by the integrin receptor signalling pathway [69]. PLD is implicated in cell spreading of ovarian cancer cells after PA was found to interact directly with Rac1 and to mediate the translocation of GTP-Rac1 to the plasma membrane [69]. This activated downstream p21-activated kinase, inducing integrin-mediated lamellipodia formation and cell spreading. A number of ovarian cancer cell lines constitutively express LPA [70]. In SKOV3 ovarian cancer cells that constitutively produced LPA, inhibition of PLD activity resulted in a 50% reduction of LPA levels [70]. Interestingly, OVCAR-3 ovarian cancer cells were shown to increase LPA production following treatment with LPA at a concentration similar to that found in patient ascites. This was reduced by 60% following the addition of an inhibitor of PLD activity [70]. These findings indicate that activity of PLD is associated with both constitutive and LPA-induced LPA production in ovarian cancer cells [70].

### 3.2. Sphingolipids

Altered sphingolipid metabolism is also associated with the pathogenesis of a variety of human malignancies and includes changes in the levels of sphingolipids and the enzymes involved in their metabolism [71]. Ceramides, short chain sphingolipids, have been most extensively studied in cancer, due to their involvement in apoptosis. Ceramides are bioactive lipids and are primarily formed by the hydrolysis of sphingomyelin, following activation of acid or neutral sphingomyelinase or by *de novo* synthesis involving N-acylation of dihydrosphingosine [72]. They are proapoptotic metabolites and known tumour-suppressors. The administration of ceramide analogues can inhibit tumour growth in a variety of cancer cell lines, both *in vitro* and *in vivo*[73–76]. Regulated by many enzymes, ceramides give rise to more complex sphingolipids, including sphingosine 1-phosphate (S1P), a bioactive lipid shown to promote cell survival. S1P is formed following activation of the enzyme sphingosine kinase (SphK). The isoform SphK1 is activated in human ovarian cancer cells, leading to resistance to the chemotherapy drug, 4-HRP [77]. The ability of S1P to facilitate the survival of cancer cells is in direct contrast to the proapoptotic activity of ceramide, and consequently, it is suggested that the ratio of these two lipids are important in cancer development [78,79].

### 3.3. Glycosphingolipids

Ceramides can also lead to the synthesis of glycosphingolipids, including sulphatides and gangliosides. Sulphatides (ST), membrane-sulphated glycolipids, have been reported to be elevated in ovarian cancer [80,81]. Microarray analysis revealed an increase in the mRNA expression of enzymes involved in ST synthesis in microdissected epithelial ovarian cancer cells, when compared to normal tissue [80]. However, their role in the pathogenesis of ovarian cancer remains to be elucidated.

## 4. Lipids as Diagnostic Markers and Therapeutic Targets in Ovarian Cancer

### 4.1. Diagnostic Markers

Lipid biomarker discovery is of particular interest in ovarian cancer, as mortality could be reduced significantly by an early detection test. However, only a few studies have been reported to date investigating lipids as diagnostic ovarian cancer markers. Some promising lipid biomarkers are shown in Table 1. Studies investigating the potential role for LPA as an early detection marker presented conflicting results. LPA levels were shown to be significantly elevated in ovarian cancer patients when compared to controls in some studies [4,41]; however, other researchers found no differences [43] or even contrary results [4]. These differing results can potentially be attributed to differing methodology and/or sample type. An ongoing clinical trial is currently validating the utility of a new assay for LPA for the early detection of ovarian cancer [82].

Analysis of EOC tissue using liquid chromatography electrospray ionization-tandem mass spectrometry (LC-ESI-MS/MS) revealed elevated levels of sulphatides (ST) when compared to normal ovarian tissue [80]. Matrix-assisted laser desorption ionization-tissue-imaging MS detected, as well as localized, ST in areas of epithelial ovarian carcinoma, further supporting the potential of ST as a biomarker in ovarian cancer. Using fluorometric assays, PLA_2_ was identified as a potential diagnostic maker after increased activity levels were observed in human EOC tissues when compared to either benign or normal tissues [44].

Lysophospholipids (LPLs) might also have potential as diagnostic and/or predictive ovarian cancer markers [3,41,83]. Elevated levels of LPA and lysophosphatidylinositol (LPI) were found in plasma of ovarian cancer patients using electrospray ionization-tandem mass spectrometry (ESI-MS) [3]. ESI-MS also identified a significant difference in plasma levels of LPA, LPI and sphingosine-1-phosphate (SIP) when comparing patient and control samples [41]. The potential value of LPLs as predictive markers arose after significant differences between levels of SIP, LPA and lysophosphatidylcholine (LPC) were identified between preoperative and postoperative serum samples [41]. The measurement of bioactive phospholipids, LPA, LPI, LPC and SIP, in conjunction with serum CA125 using LC ESI-MS/MS improved the accuracy of diagnosing early stage serous ovarian cancers from 65% to 82% and mucinous cancers from 44% to 88% [83].

### 4.2. Therapeutic Targets

Lipogenic enzymes and lipids are increasingly seen as potential targets of novel anti-ovarian cancer therapies. Potential components of aberrant metabolic lipid pathways, which might serve as targets to inhibit cancer growth and/or to overcome chemotherapy resistance, are listed in Table 2.

Statins are inhibitors of cholesterol synthesis, but also of the production of molecules, such as isoprenoids. These are lipid anchors for a range of signalling proteins, such as the small GTPases, Ras and Rho [84]. The statin lovastatin has recently been reported to induce apoptosis of ovarian cancer cells in a p53-independent manner and to synergize with doxorubicin, a chemotherapeutic agent used to treat recurrent ovarian cancer. Lovastatin drives ovarian tumour cell death by two mechanisms: first, by blocking HMG-CoA reductase activity, and second, by sensitizing multi-drug resistant cells to doxorubicin by a novel mevalonate-independent mechanism [85]. The efficacy of statins in anti-ovarian cancer therapy is currently examined in a phase II clinical study of interaction of lovastatin and paclitaxel for patients with refractory or relapsed ovarian cancer [86].

To overcome chemotherapy resistance, the effects of co-administration of ceramide with chemotherapy agents has been investigated. In human ovarian cancer cells, the co-administration of ceramide with the chemotherapy drug paclitaxel sensitized the cancer cells and doubled cell death when compared to treatment of ovarian cancer cells with paclitaxel alone [87]. In addition, following paclitaxel treatment, the expression of ceramide transport protein (CERT) was increased in chemotherapy resistant cell lines and in tumour samples of ovarian cancer patients [88]. CERT transports ceramide generated by *de novo* synthesis from the ER to the Golgi apparatus, where it is converted to sphingomyelin [89]. Knockdown of CERT resulted in an accumulation of ceramide in the ER, increasing ER stress and sensitivity of cancer cells to paclitaxel treatment [88]. Consequently, ceramide has also become a therapeutic target in ovarian cancer.

The FAS inhibitor, C93, has been shown to greatly increase the cytotoxicity of the chemotherapy drugs carboplatin and paclitaxel in ovarian cancer cells [25], thereby highlighting the potential of this inhibitor as a drug to overcome chemotherapy resistance and disease recurrence. A more recently identified inhibitor of FAS, P75, significantly reduced cell proliferation in ovarian clear cell carcinoma [30] and decreased cell proliferation and induced apoptosis in ovarian cancer cells [31].

The gene ectonucleotide, pyrophosphatase/phosphodiesterase 2 (ENPP2), which encodes ATX, was identified as a potential gene causing platinum-resistance in ovarian cancer [90]. Inhibition of ENPP2 and ATX using siRNA and 2-carbacyclic phosphatidic acid, respectively, led to increased carboplatin-induced apoptosis of ovarian cancer cells. In addition, expression of ATX in the ovarian cancer cell line, OVCAR-3, delayed apoptosis, suggesting that ATX inhibits cell death induced by the chemotherapy drug carboplatin [90].

## 5. Conclusions

Abnormal lipid metabolism plays an important role in the pathogenesis of ovarian cancer. The activity of lipid metabolizing enzymes is regulated by a complex interplay between metabolic and oncogenic signalling pathways. Components of the aberrant lipid metabolism pathways are promising targets for the development of novel ovarian cancer diagnostics and therapeutics. Several diagnostic markers with potential for the early detection of ovarian cancer have been identified. LPA and LPLs show the most promise, with elevated plasma levels identified in ovarian cancer patients. Therapeutic targets that have shown the greatest potential include statins, as well as ceramides. Both statins and ceramides are successful in inducing ovarian cancer cell death, as well as sensitising cancer cells to chemotherapeutic drugs. FAS inhibitors, C93 and C75, which inhibit the growth and survival of ovarian cancer cells, also show promise. LPA and statins are currently under investigation in clinical trials for their potential in the detection and treatment of ovarian cancer, respectively. This review highlights the important role of abnormal lipid metabolism in the development and progression of ovarian cancer.

## Figures and Tables

**Table 1:** Altered lipid metabolism in ovarian cancer: potential diagnostic markers.

Marker	Studies/Methods	References
LPA	LPA assays under development in clinical trials.	[82]

Sulphatides	Elevation of sulphatides in EOC tissue compared to normal tissue by mass spectrometry (LC ESI-MS/MS).	[80]

PLA_2_	Elevated activity of PLA_2_ in EOC tissue, compared to benign tumours and normal tissue using fluorometric assays.	[44]

LPLs	Increased levels of LPA, LPI and SIP in plasma from ovarian cancer patients compared to healthy controls using ESI-MS.	[3,41]
	Predictive markers with a significant difference between levels of SIP, LPA and LPC between preoperative and postoperative ovarian cancer patients.	[41]

LPLs + CA 125	Serum measurements of LPLs in conjunction with CA125 greatly improved diagnostic accuracy in early stage serous ovarian cancer and mucinous subtypes using LC ESI-MS/MS.	[83]

Abbreviations: PLA_2_, phospholipase A_2_; LPLs, lysophospholipids; LPA, lysophosphatidic acid; LPI, lysophosphatidylinositol; LPC, Lysophosphatidylcholine; SIP, sphingosine-1-phosphate; LC ESI-MS/MS, liquid chromatography electrospray ionization-tandem mass spectrometry; ESI-MS, electrospray ionization mass spectrometry.

**Table 2:** Altered lipid metabolism in ovarian cancer: potential therapeutic targets.

Target	Treatment	Effect	References
Cholesterol	Statins	Induce apoptosis of ovarian cancer cells. In clinical trials.	[85,86]
FAS	C93	Increases cytotoxicity of carboplatin and paclitaxel. Induces cell death *in vitro* and reduces tumour growth in xenograft mouse model.	[25,29]
	C75	Reduces cell proliferation in ovarian clear cell carcinoma cell lines. Inhibits growth and survival of ovarian cancer cells *in vitro*.	[30,31]
ATX	ccPA	Increases cytotoxicity of carboplatin.	[90]
ENPP2	siRNA	Increases apoptosis of cancer cells with carboplatin.	[90]
Ceramide	Ceramide	Co-administration with the chemotherapy drugs, paclitaxel or carboplatin, sensitises cancer cells, increasing cell death.	[87]
CERT	siRNA	Sensitises cancer cells to paclitaxel.	[88]

Abbreviations: Fas, fatty acid synthase; ATX, autotaxin; ccPA, 2-carbacyclic phosphatidic acid; ENPP2, ectonucleotide pyrophosphatase/phosphodiesterase 2; CERT, ceramide transport protein.
